# Oxymatrine alleviates symptoms in high-fat diet and STZ-induced SD rats with painful diabetic neuropathy by reducing inflammation and oxidative stress

**DOI:** 10.3389/fimmu.2026.1750051

**Published:** 2026-02-17

**Authors:** Zhi Ming, Yuning Liu, Shuaiying Jia, Yao Su, Wei Yan, Jingyan Lin

**Affiliations:** 1 Department of Anesthesiology, The Affiliated Hospital of North Sichuan Medical College, Nanchong, China; 2 Department of Medical Imaging, The Affiliated Hospital of North Sichuan Medical College, Nanchong, China

**Keywords:** aquaporin 4, glymphatic system, inflammation, oxidative stress, oxymatrine, painful diabetic neuropathy

## Abstract

**Introduction:**

Painful Diabetic Neuropathy (PDN) is a severe complication of diabetes, featured by intricate aetiology and multiple side effects of current therapeutic approaches. In recent years, the glymphatic system has attracted increasing attention for its role in PDN. This study investigated the regulatory effects and underlying mechanisms of Oxymatrine (OMT) on the spinal glymphatic system in PDN rat models, aiming to provide novel therapeutic insights for PDN.

**Methods:**

The PDN rat model was established by high-fat and high-sugar diet combined with streptozotocin (STZ) induction. The 50% paw withdrawal threshold (50% PWT) was measured by Von Frey filaments to evaluate neuropathic pain. Spinal glymphatic system function was observed via Magnetic Resonance Imaging (MRI). Western blotting was used to detect the expression of Aquaporin-4 (AQP-4), Metalloproteinase-9 (MMP-9), NF-κB p65, p-p65, Nrf2 and HO-1. Immunofluorescence was performed to assess AQP4 polarization and nuclear expression of p65. In addition, the levels of oxidative stress indicators (GSH, SOD, MDA) and inflammatory factors (IL-1β, IL-6, TNF-α) were determined.

**Results:**

OMT treatment significantly alleviated PDN-related symptoms and improved the detected indicators. It effectively reduced oxidative stress and inflammatory levels, upregulated the expression of Nrf2 and HO-1, downregulated MMP-9 expression, repaired AQP-4 polarisation, and restored the function of the spinal glymphatic system in PDN rats.

**Discussion:**

This study provides a theoretical foundation for the potential application of OMT as a therapeutic agent for PDN, and its multi-target regulatory mechanism offers new directions for PDN treatment.

## 1 Introduction

Diabetes, a chronic metabolic disease with a high global incidence, has witnessed a significant increases in both prevalence and mortality in recent years. According to the latest statistics, the global diabetic population reached 529 million in 2021 and is projected to increase to 1.31 billion by 2050 (1). This distressing trend has imposed a heavy economic and lifestyle burden on individuals, families, and society (2). Among diabetic patients, 20% to 30% are likely to progress to Painful Diabetic Neuropathy (PDN) (3), Common clinical manifestations include intense foot pain, burning sensations, or needle-like pain. Some patients may also experience cold pain, itching, numbness, and abnormal sensations in response to touch or temperature changes (4). These symptoms not only cause physical and mental suffering but also often accompany issues like anxiety, depression, and sleep disorders, which can significantly reduce the patients’ quality of life (5). Although the mainstay treatments for PDN include tricyclic antidepressants, duloxetine, pregabalin, gabapentin, etc. (6), these medications are frequently accompanied by various side effects (7, 8) that can lower treatment compliance and further impact the patients’ quality of life. Consequently, in-depth research on the pathogenesis of PDN and exploration of novel and effective treatments have become pivotal directions in current clinical research.

In recent years, as research on the glymphatic system has deepened, its role in PDN has gradually been recognized (9). The glymphatic system, which is in charge of clearing metabolic waste and preserving cerebral fluid homeostasis within the central nervous system (10, 11), is primarily composed of perarterial spaces, perivenous spaces, and astrocyte end-feet (12). Its key process involves cerebrospinal fluid (CSF) flowing from the subarachnoid space into the brain tissue through perarterial spaces, exchanging substances with the interstitial fluid, and then carrying metabolic waste out along perivenous spaces to be drained into cervical lymph nodes through meningeal lymphatic vessels (13). This process is vital importance for eliminating metabolic waste and maintaining cerebral fluid balance, thereby providing a stable microenvironment for the normal function of central nerve cells (14). The proper functioning of the glymphatic system relies on the polarized distribution of AQP4, which is correctly located on astrocyte end-feet rather than in the cell body or other locations (15). Previous studies have indicated that the polarized distribution of AQP4 depends on the dystrophin glycoprotein complex (DGC) (16). The DGC’s core components include dystrophin, β-Dystroglycan (β-DG), and other related proteins. Within the DGC, the C-terminal cytoplasmic domain of β-DG interacts with dystrophin to form a stable mechanical link, connecting the extracellular matrix with the intracellular cytoskeleton (17). This connection not only offers structural support to the cell membrane but also ensures the proper polarized expression of AQP4 on astrocyte end-feet.

Oxidative stress (OS) is a state of imbalance between oxidation and antioxidants in the body, characterized by excessive production of reactive oxygen species (ROS) and other reactive molecules, or a reduced capacity of the body to clear these oxidative molecules (18). Nrf2 is the core transcription factor mediating the body’s antioxidant stress response, and its functional integrity is crucial for inhibiting OS-induced damage and maintaining normal physiological functions of tissue cells (19). Under physiological conditions, Nrf2 binds to Keap1 in the cytoplasm to form a complex, and remains in a resting state (20). When the body is in an OS state, the generated ROS can disrupt the binding between Nrf2 and Keap1, prompting Nrf2 to be released from the complex and undergo nuclear translocation, subsequently initiating the transcription and expression of a variety of downstream antioxidant genes (21), among which HO-1 is a key downstream target gene. HO-1 exerts potent antioxidant, anti-inflammatory, and cytoprotective effects by catalysing the production of relevant active products. However, when the intensity of OS in the body exceeds its compensatory capacity, the antioxidant effect of the Nrf2-HO-1 pathway becomes decompensated (22). At this point, excessive ROS are difficult to be effectively cleared, leading to their continuous accumulation in cells and tissue microenvironment, ultimately resulting in damage. Excessive ROS can impair the function of the glymphatic system through various mechanisms. On one hand, elevated levels of ROS can damage IκB proteins, leading to their degradation and the release of NF-κB. Once NF-κB enters the nucleus, it binds to the promoter regions of various pro-inflammatory factor genes, promoting the transcription and expression of these genes, increasing the synthesis and secretion of pro-inflammatory factors, and thereby further exacerbating the inflammatory response (23, 24). The increased levels of pro-inflammatory factors can then stimulate the elevation of OS levels through multiple pathways, creating a vicious cycle that ultimately leads to cellular dysfunction and tissue damage. On the other hand, ROS directly disrupts the central neurotransmitter metabolic balance and exacerbate glymphatic system dysfunction (25). OS can also impact AQP4 expression through multiple pathways: 1) ROS activate signalling pathways such as MAPK and NF-κB, leading to upregulated AQP4 expression (26), which contributes to brain oedema (27). 2) During telestroke, ROS enhance AQP4 membrane expression through Src kinase-mediated Cav1 Y14 phosphorylation, resulting in brain oedema (28); 3) ROS activate HIF - 1α, which binds to the AQP4 gene promoter region to promote AQP4 transcription and translation (29), thereby increasing AQP4 expression. However, it remains unclear whether OS and elevated levels of inflammation can alter the polarized distribution of AQP4, and thereby cause dysfunction of the spinal cord glymphatic system.

In various neurological diseases, including Parkinson’s disease (30), Alzheimer’s disease (31), and cerebral oedema (32), glymphatic system dysfunction is closely associated with elevated Matrix Metalloproteinase-9 (MMP-9) expression. MMP-9 specifically cleaves the extracellular domain of β-DG, disrupting its connection with the basement membrane (33). This compromises the structural integrity of the DGC, causing it to lose the AQP4 anchoring function. Consequently, AQP4 polarization is disrupted, leading to glymphatic system dysfunction and hindering the normal exchange of cerebrospinal fluid (CSF) and interstitial fluid (ISF). The resulting accumulation of central ROS and inflammatory factors (34) affects CNS stability and function, creating a vicious cycle. Therefore, we speculate that the combined effects of OS and inflammatory factors can regulate the expression of MMP-9, disrupt AQP4 polarization, and cause glymphatic dysfunction, which may be a potential strategy for the treatment of PDN.

Oxymatrine (OMT) is an alkaloid extracted from the desiccated roots of Sophora flavescens. Research has confirmed its remarkable anti-inflammatory and antioxidant capacity in various biological systems (35). OMT can scavenge ROS and modulates antioxidant enzyme activity. In addition, it inhibits the release of pro-inflammatory factors and regulates inflammatory signalling pathways, thus demonstrating potent antioxidant and anti-inflammatory properties (36). Moreover, OMT has shown significant therapeutic potential in a number of diseases, including cardiovascular diseases (37) and osteoarthritis (38). However, the efficacy and potential mechanisms of OMT in PDN have not been fully elucidated. Therefore, this study focuses on the relationship between OS and inflammatory factors with the polarization of AQP4, exploring whether OMT can promote the activation of the Nrf2 pathway, reduce OS levels in PDN rats, enhance the body’s antioxidant capacity, simultaneously inhibit the NF-κB signalling pathway to reduce inflammation levels, regulate the expression of MMP-9, repair the polarization of AQP4, improve the function of the glymphatic system in the spinal cord of PDN rats, and alleviate neuropathic pain symptoms in PDN rats through multiple pathways.

## 2 Materials and methods

### 2.1 Animals

This study used 70 adult male SD rats, aged 6~8 weeks and weighing 180~200g, as experimental subjects. During the experiment, three rats were housed per cage. The environmental conditions were maintained at 24°C ± 1°C with 50% ± 5% humidity and a 12-h light-dark cycle. The experimental animals had free access to food and water. All animals were purchased from the Sichuan Provincial Laboratory Animal Center and were raised in the Laboratory Animal Facility at North Sichuan Medical College. All experimental protocols were approved by the Ethics Committee of North Sichuan Medical College (authorization number: NSMC2025064) and strictly followed the Guidelines for the Care and Use of Laboratory Animals.

In this study, a type 2 diabetes rat model with insulin resistance was established by injecting low-dose streptozotocin (STZ) combined with a high-fat diet (39). This model more accurately mimics the insulin-resistant state of type 2 diabetes and can stably induce typical PDN symptoms in about 50% of rats (40).

The entire experimental period was 16 weeks, during which blood glucose and body weight of rats were regularly measured on a weekly basis. In the first week, all rats underwent adaptive feeding. Subsequently, all animals were randomly divided into the control group (The group C, 10 rats) and the model group (The group M, 60 rats). From the second week onwards, the group C were fed with a standard diet, while the group M were fed with 45% high-fat diet (HF45, Dyets Biotechnology (Wuxi) Co., Ltd., Anhui, China).

### 2.2 Establishment of PDN models and drug administration

**Figure 1:**
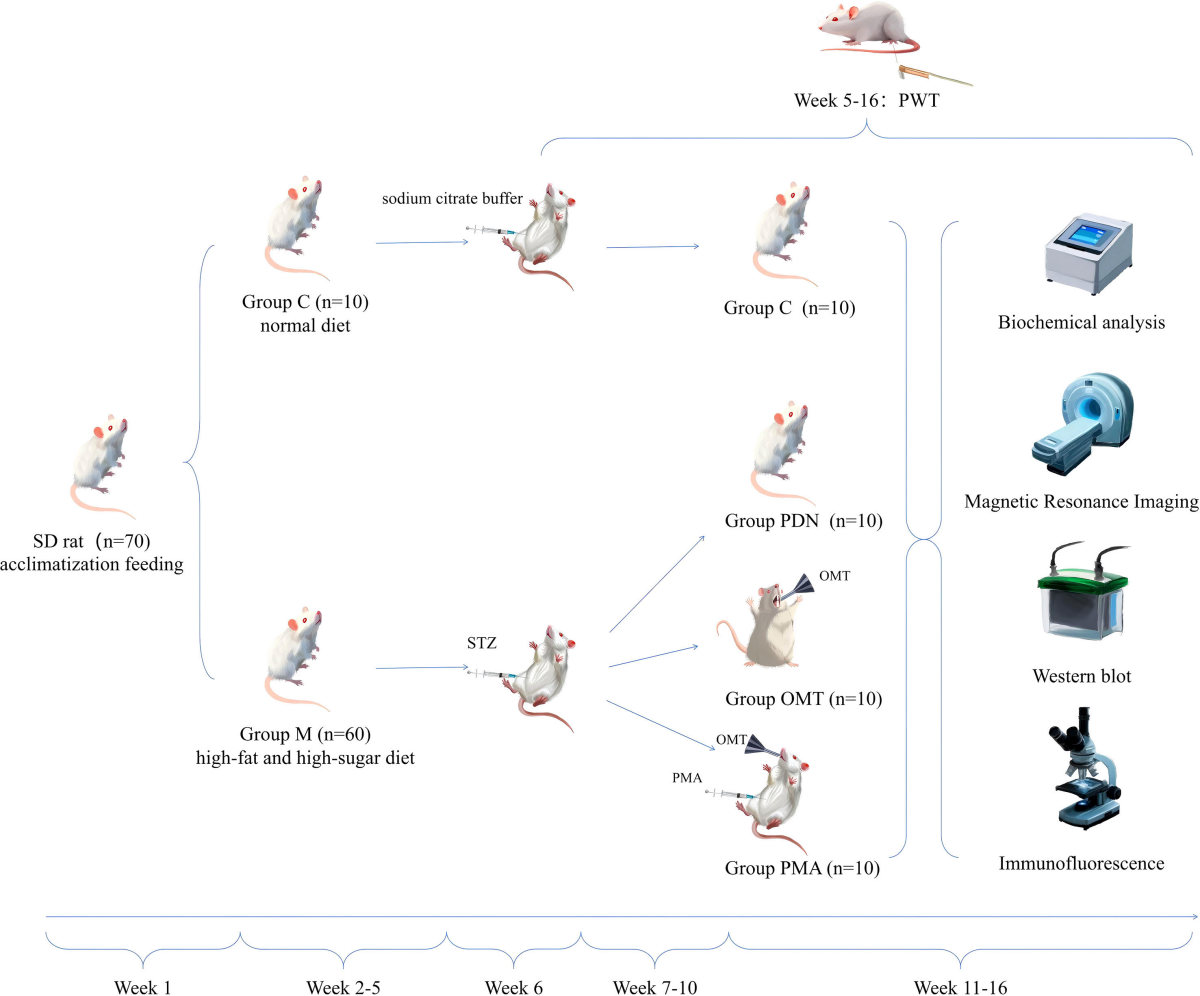
Experimental flowchart. From week 5, M group rats received intraperitoneal STZ injections, while C group rats received equal-volume citrate buffer. After PDN model establishment, the OMT group was gavaged with oxymatrine daily, and The PMA group received intragastric OMT and weekly intraperitoneal Phorbol-12-Myristate-13-Acetate(PMA). The C and PDN groups were gavaged with normal saline. The OMT and PMA groups were treated for 6 weeks. Then, 3 rats per group underwent MRI. Post-MRI, spinal cords were extracted for Western Blot analysis. Of the remaining 7 rats, 4 were used for antioxidant enzyme assays, and 3 for immunofluorescence and ROS probe detection.

In week 6, the group M was injected intraperitoneally with STZ (35 mg/kg, 1 mL/kg, HY-13753, MedChemExpress, Shanghai, China) to induce diabetes, while the group C was treated with the same volume of 10 mmol/L citrate buffer (pH 4.5). After 24 hours, blood glucose levels were measured in all rats after fasting. When the fasting blood glucose level was ≥11.1 mmol/L for two consecutive times (41), type 2 diabetes induction was considered successful. From week 7, 50% paw withdrawal threshold (50% PWT) testing was performed on diabetic rats to identify those with typical PDN. In week 10, rats meeting the PDN criteria were randomly divided into three groups (n=10): the group PDN received daily intragastric administration of saline; the group OMT was given Oxymatrine (16837-52-8, 120 mg/kg, 1 mL/kg, Chengdu Alfa Biotechnology Co., Ltd., Sichuan, China) daily for six weeks; and the group PMA received OMT intragastrically daily and intraperitoneal PMA (500 μg/kg, 1mL/kg, MedChemExpress, Shanghai, China, HY-18739) weekly for six weeks. This treatment was designed to activate the NF-κB pathway to block the therapeutic effect of OMT against PDN mediated through the NF-κB signalling pathway, thereby verifying the core mediating role of the NF-κB signalling pathway in the effect of OMT against PDN. Rats that did not meet the PDN criteria were euthanized by excess anaesthesia until cardiac arrest. Before the experimental endpoint, all rats were anesthetized via intraperitoneal injection of tribromoethanol (HY-B1372, 240 mg/kg, 40 mg/ml, MedChemexpress, Shanghai, China). The experimental procedure is shown in **Figure 1**.

### 2.3 50% Mechanical paw withdrawal threshold

In this study, the 50% PWT was adopted as the primary indicator to assess neuropathic pain, which can more accurately reflect the mechanical sensitivity changes in rats and reduce experimental errors compared with PWT. Starting from week 6, a trained and designated experimenter used Von Frey filaments (US PAT 58239698512259, North Coast, CA, USA) to test the hind paw pain threshold of rats weekly. Before the experiment, rats were placed in a metal mesh device for 30 minutes to acclimate. During testing, Von Frey filaments were pressed vertically against the middle of the hind paw for 5 seconds each time, with intervals of approximately 30 seconds. A positive response was recorded when the rat exhibited paw withdrawal or licking behaviour (42). Each hind paw of the rats was tested at five different points, and the stimulus intensity that elicited a 50% probability of PWT was calculated using the “up-down method”. To avoid foot injury, all procedures were strictly performed in accordance with the instrument operation specifications, with the maximum press intensity not exceeding 26g. To rule out the impact of circadian rhythms, all tests were carried out between 9:00 and 12:00 a.m. (43).

### 2.4 Magnetic resonance imaging measurement

At the end of the treatment period, three rats were randomly selected from each group for imaging assessment. All rats underwent MRI scanning at the MRI laboratory of the Affiliated Hospital of North Sichuan Medical College (Wenhua Road Campus) using a 3.0T clinical magnetic resonance scanner (Discovery MR750, GE Healthcare, USA). To minimize the impact of anaesthetic drugs on the central metabolism of rats (44), all rats were anesthetized with sevoflurane, which has a faster metabolism (45), at an induction concentration of 2.5%~3.0%. After anaesthesia induction, the rats were secured in a custom eight-channel rat coil. The scanning focused on the first lumbar vertebra of the rats, extending 3 cm above and below, and T1-weighted images were acquired using a fast spin-echo sequence to observe the dynamic changes of the contrast agent in the spinal cord.

The scan parameters were as follows: Time of Repetition (TR) 450.0 ms, Time to Echo (TE) 5.4 ms, slice thickness 2.0 mm, slice space 1.0 mm, Field of View (FOV) 6.5 cm×6.5 cm, matrix size 256×256, flip angle 90°, bandwidth 31.25 kHz, and frequency encoding direction Field of View (A/P). All scan parameters were kept consistent to ensure data reproducibility.

After the initial image scanning, the rats were fixed on the operating table and injected with a 10% Gd-DTPA solution (100 μL Gd-DTPA+900 μL 0.9% saline) at the L4/5 segment via a micro-injector at a rate of 5 μL/min, with a total volume of 25 μL. Post injection, MRI scans were performed at 5, 15, 30 minutes and 1, 2, 3, and 6 hours to measure the dynamic changes of the contrast agent in the spinal cord. During the experiment, the rats’ vital signs were monitored in real-time. An air heating system was used to maintain a stable body temperature, ensuring normal heart rate and body temperature to prevent adverse reactions (46). The bladder was manually pressed to assist urination during anaesthesia, and rats were allowed to drink water freely between scans. After all imaging was completed, the rats were euthanized under deep anaesthesia, and tissue samples were immediately taken for further analysis.

### 2.5 Western blot

Rat spinal cord tissue was placed in a 1.5 mL grinding tube with RIPA lysis buffer containing protease and phosphatase inhibitors, plus 3 mm grinding beads. It was ground twice for 90 seconds each at -20 °C, then homogenized at 4 °C for 30 minutes. After two centrifugation (4 °C, 12000 r/min, 10 minutes each), the supernatant was collected. Protein concentration was measured using a BCA kit (Beyotime Biotechnology, Shanghai, China, Cat. No. P0010) with all steps performed on ice.

After electrophoresis, proteins were transferred to a PVDF membrane treated with formaldehyde. The membrane was blocked with protein-free rapid blocking solution and incubated with primary antibodies MMP-9 (1:2000, Proteintech Group, Inc, Wuhan, China), GFAP (1:5000, Proteintech Group, Inc, Wuhan, China), AQP4, NF-κB p65/p-p65, Nrf2, HO-1 (1:1000, Abmart Shanghai Co.,Ltd., Shanghai, China) overnight at 4 °C. After washing with TBST, it was incubated with secondary antibody (goat anti - rabbit/mouse IgG, 1:15000, Proteintech Group, Inc, China) for 2 h at room temperature. α-tubulin or β-actin (1:10000, Proteintech Group, Inc, China) served as loading controls. Finally, target protein IOD was measured using a gel imager (ChemiDoc XR, Bio-Rad, USA).

### 2.6 Biochemical analysis

Four rats were randomly selected from each group, deeply anesthetized, and perfused with 0.9% saline via cardiac puncture. The spinal cord tissue from the L1-L3 segments was excised and placed in 1.5 mL grinding tubes containing two 3 mm beads along with an appropriate volume of tissue lysis buffer or PBS. The samples were then homogenized using a tissue homogenizer. After homogenization, the samples were incubated on ice for 10 minutes to facilitate lysis and subsequently centrifuged at 10,000 rpm for 10 minutes. The resulting supernatants were carefully collected for further analysis.

OS indicators were measured using the Total Superoxide Dismutase Assay Kit with WST-8 (S0101S, Beyotime Biotechnology, Shanghai, China), the GSH and GSSG Assay Kit (S0053, Beyotime Biotechnology, Shanghai, China), and the Lipid Peroxidation MDA Assay Kit (S0131S, Beyotime Biotechnology, Shanghai, China). Inflammatory factors levels were measured using the Rat IL-1β ELISA Kit (PI303, Beyotime Biotechnology, Shanghai, China), the Rat IL-6 ELISA Kit (PI328, Beyotime Biotechnology, Shanghai, China), and the Rat TNF-α ELISA Kit (PT516, Beyotime Biotechnology, Shanghai, China). All measurements were performed on a SpectraMax iD3 multimode reader (Meggitt Molecular Instruments (Shanghai) Co., Ltd.).

### 2.7 Fluorescent chemical method and immunofluorescence

Three rats were randomly selected, deeply anesthetized, and then perfused through the heart with 0.9% normal saline followed by 4% paraformaldehyde. The L1-L3 spinal cord segments were removed and fixed in 4% paraformaldehyde, and then 3 μm thick paraffin sections were prepared. Another portion of the same spinal cord segments was embedded in OCT compound, frozen in liquid nitrogen, and then cut into 7 μm thick sections using a cryostat at -20 °C for immediate use in subsequent experiments. For paraffin sections, one group was co - stained with AQP4 (1:400, Abcam plc, Cambridge, UK) and GFAP (1:200, Proteintech Group, Inc, Wuhan, China), while another group was co-stained with NF-κB p65 (1:200, Bioss, Beijing, China) and MMP-9 (1:200, Proteintech Group, Inc, Wuhan, China). After primary antibody incubation, sections were incubated with fluorescent secondary antibodies and counterstained with DAPI. Coverslips were mounted using an anti-fade reagent. For frozen sections, ROS levels were assessed using DHE (1:200, S0063, Beyotime Biotechnology, shanghai, China) by incubating at 37 °C for 30 minutes in the dark, followed by DAPI counterstaining and mounting with an anti-fade reagent. All sections were observed under an Olympus FV1200 confocal laser scanning microscope. To ensure reproducibility, laser power, pinhole size, and imaging parameters were kept consistent across all samples. Three random images were taken from each section in the anterior horn, posterior horn, and central canal regions of the spinal cord. Image analysis was performed by blinded personnel and included assessment of AQP4 polarization, astrocyte morphology analysis, NF-κB p65 nuclear translocation rate, and ROS fluorescence intensity.

To quantify AQP4 polarization, this study analysed the AQP4 polarity rate based on the Donut method (33). The steps were as follows: First, using ImageJ software, a region of interest for the periastracyte space (ROI_PAS_)was selected by expanding 5 pixels outward from the GFAP fluorescence range, and the AQP4 fluorescence intensity within this region was measured. Then, a region of interest for the astrocyte cell body space (ROI_ASS_) was selected, and its AQP4 fluorescence intensity was measured. The AQP4 fluorescence intensity in ROIPAS was subtracted from that in ROIASS, and the result was divided by the global AQP4 fluorescence intensity in the entire image (ROI_global_) to calculate the polarization rate of normally polarized AQP4. The formula was:


$$\begin{array}{c} AQP4\;polarization\;rate\\ =\frac{ROI_{PAS}\;AQP4\;fluorescence\;intensity-ROI_{ASS}\;AQP4\;fluorescence\;intensity}{ROI_{global}\;AQP4\;fluorescence\;intensity} \end{array}$$

For each section, the AQP4 polarization rate was calculated from three images using the formula, and the average value was taken as the sample’s AQP4 polarization rate. To quantitatively assess the morphological characteristics of astrocytes, we employed Sholl analysis (47). The specific procedure is as follows: First, five astrocytes stained with GFAP were randomly selected from each section for analysis. With the cell nucleus labelled by DAPI as the centre, a series of concentric circles with an initial radius of 5 μm and an interval of 2 μm were drawn until the farthest end of the cell protrusion was covered. Finally, the intersection positions and numbers between these concentric circles and astrocyte protrusions were recorded in detail for analysis. All image analyses were performed using ImageJ software (version 2022, National Institutes of Health).

### 2.8 Network pharmacology analysis

#### 2.8.1 Acquisition and collation of targets

Retrieve OMT from the PubChem database (https://pubchem.ncbi.nlm.nih.gov/), extract its Isomeric SMILES and record it; subsequently, import the Isomeric SMILES into the SEA database (https://sea.bkslab.org/), CTD database (https://ctdbase.org/), and SWISS Target Prediction database (http://www.swisstargetprediction.ch/) respectively to jointly predict the potential therapeutic targets of OMT, and merge the prediction results from the three databases. Using “diabetic neuropathy” as the search keyword, obtain 2343 potentially relevant targets from the GenGards database and 123 related targets from the OMIM database. Merge the targets from the two databases and remove duplicates, ultimately obtaining 2371 core targets related to diabetic neuropathy.

#### 2.8.2 Construction of networks and functional enrichment analysis

Use the online Venn diagram visualization tool jvenn (https://www.bioinformatics.com.cn/static/others/jvenn/example.html) to analyse the intersection relationship between the potential targets of OMT and the core targets related to diabetic neuropathy; upload the obtained intersection targets to the String database (https://string-db.org/), set a confidence threshold ≥ 0.4 to screen for reliable protein-protein interaction relationships, and import the screened interaction data into Cytoscape 3.9.1 software to construct the PPI network; finally, perform GO functional enrichment analysis and KEGG pathway enrichment analysis on the intersection targets of OMT and diabetic neuropathy via the online tool (https://www.bioinformatics.com.cn/static/others/jvenn/example.html).

### 2.9 Statistical analysis

All data were analysed using GraphPad Prism 9.5 (GraphPad Software, San Diego, CA, USA). Data are presented as mean ± SD, with 95% confidence intervals also reported. Group mean comparisons used One-way analysis of variance (ANOVA). For repeated-measures data(e.g., weight, blood glucose, PWT), time-point differences were assessed with repeated-measures ANOVA. All *post-hoc* multiple comparisons were subjected to Bonferroni correction to control the Type I error rate. Specifically, one-way ANOVA applied Bonferroni adjustment to all pairwise comparisons, whereas two-way ANOVA employed the correction only for comparisons among different groups at the same time point. Significance was set at P<0.05.

## 3 Results

### 3.1 Physiological and metabolic parameters in each group of rats

**Figure 2:**
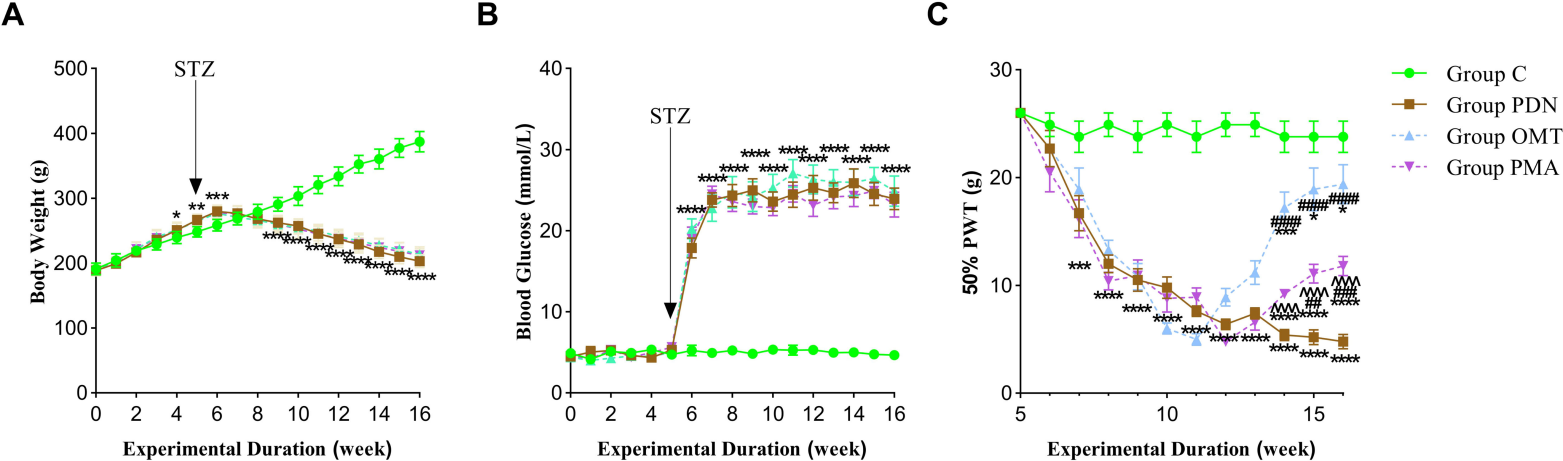
Trends in body weight, blood glucose, and PWT across groups. **(A)** Trends in body weight for the group C, PDN, group OMT, and group PMA. **(B)** Trends in fasting blood glucose for the group C, PDN, group OMT, and group PMA. **(C)** Trends in 50% PWT for the group C, PDN, group OMT, and group PMA. Comparisons among groups were performed using two-way ANOVA, with *post-hoc* analyses conducted using the Bonferroni correction method. Data are presented as mean ± SD. *versus Group C; # versus Group PDN; ∧ versus Group OMT. *#∧, P<0.05; **##∧∧, P<0.01; ***###∧∧∧, P<0.001; ****####∧∧∧∧, P<0.0001.

Throughout the experiment, weekly measurements of body weight, blood glucose, and PWT were taken to assess the PDN rat model and the impact of interventions on their physiological and metabolic state. Initially, no significant differences in these parameters were found between groups. Specific changes are shown in **Figure 2**.

#### 3.1.1 Body weight changes

During the entire experimental period, rats in the group C showed a steady weight gain. Rats in the three groups (PDN, OMT, and PMA) gained weight rapidly during the high-fat diet period (*P* = 0.0015, 95% CI=[-31.13, -5.066]). However, after STZ injection in week 5, their weights started to drop continuously from week 6 (*P* < 0.001, 95% CI=[-34.23, -8.166]). Compared with the group PDN, no significant differences in body weight changes were found in the groups OMT and PMA.

#### 3.1.2 Blood glucose levels

After STZ injection in week 5, fasting blood glucose levels in the three groups (PDN, OMT, and PMA) increased significantly and remained at or above 16.7 mmol/L [*P* < 0.001, 95% CI=(-16.19, -9.085)]. Compared to group PDN, no significant differences in fasting blood glucose levels were found in the groups OMT and PMA. This indicates that OMT does not regulate blood glucose levels in rats.

#### 3.1.3 50% PWT levels

From week 6, 50% PWT values in diabetic rats began to decline, stabilizing below 10g by week 10, confirming successful PDN model establishment. In the group OMT, which received oral administration from week 10, 50% PWT values gradually increased from week 11. The group PMA, which started OMT oral administration and weekly PMA injections from week 10, saw 50% PWT values rise from week 13. By week 16, both the groups OMT and PMA had significantly higher 50% PWT values than the group PDN (*P* < 0.001, 95% CI=[-18.93, -10.27]). However, the group OMT showed a significantly better treatment effect and earlier onset of action compared to the group PMA.

### 3.2 Functional changes in the spinal cord glymphatic system across groups

This study explored whether spinal glymphatic system dysfunction exists in PDN rats and OMT impact on it. Using Gd-DTPA-enhanced MRI, it assessed the glymphatic function in rats of different groups. The experiment selected three spinal segments: the one with the strongest signal in the lumbar enlargement region and the two adjacent ones. RadiAnt DICOM Viewer software (64-bit, 2023) was used to analyse signal intensity (SI). A blinded operator randomly selected three 0.005 cm² regions of interest (ROIs) in the anterior horn, posterior horn, and central canal of each image. The average of three SI measurements was taken, and the final data for each time point was the average SI of three segments.

**Figure 3:**
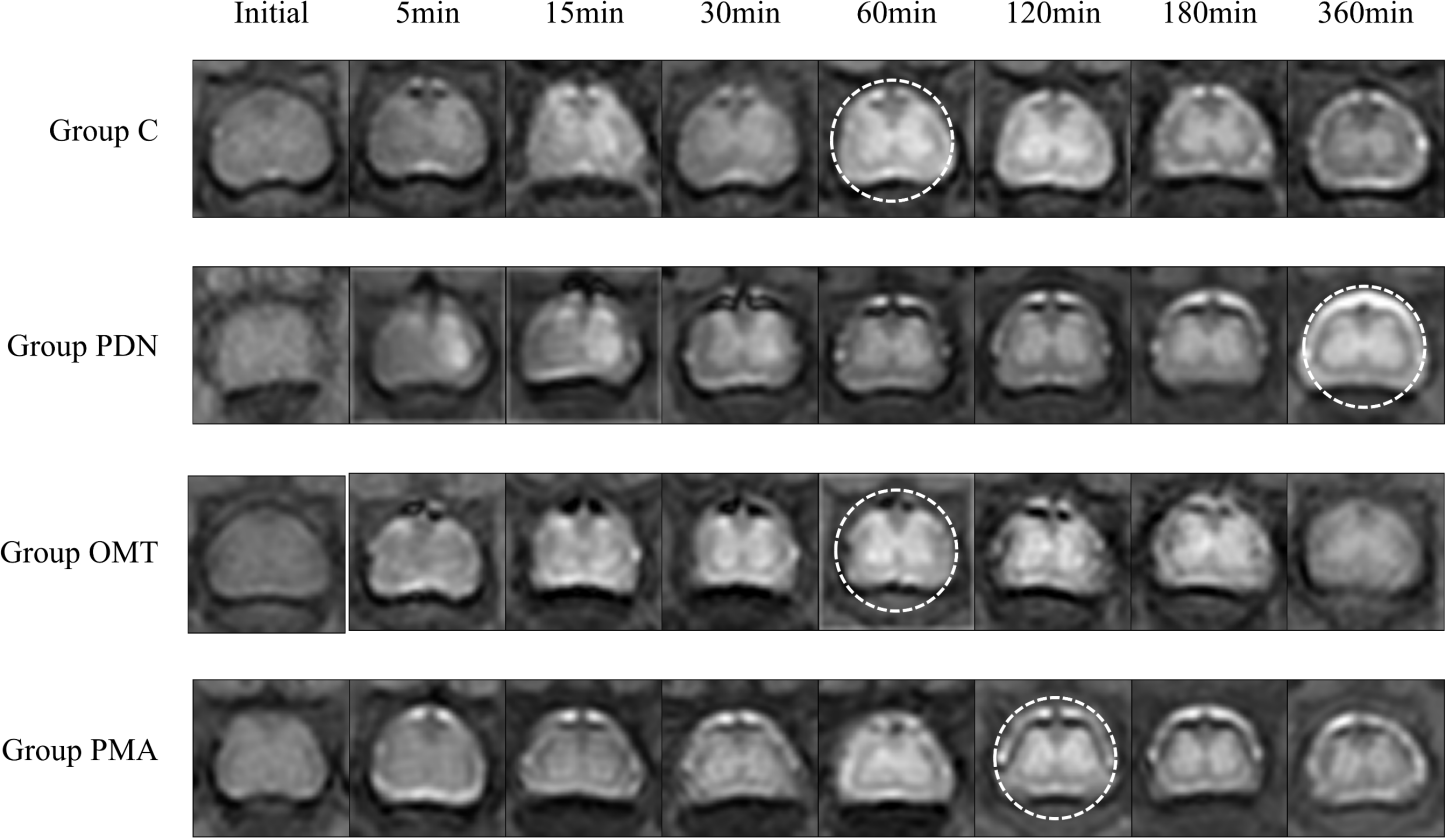
Changes in the spinal cord of rats in each group after injection. It shows the metabolic process of Gd-DTPA in the spinal cord of rats in each group. The labelled time points are as follows: Initial (before injection of Gd-DTPA), 5 min (5 minutes after injection of Gd-DTPA), 15 min (15 minutes after injection of Gd-DTPA), 30 min (30 minutes after injection of Gd-DTPA), 60 min (60 minutes after injection of Gd-DTPA), 120 min (120 minutes after injection of Gd-DTPA), 180 min (180 minutes after injection of Gd-DTPA), and 360 min (360 minutes after injection of Gd-DTPA). The circular marks in the figure represent the peak time points of MRI SI in each group of rats.

In **Figure 3**, the time to peak SI of the contrast agent in the group PDN was significantly delayed, with the “butterfly sign” appearing latest, indicating reduced absorption (*P* = 0.0353, 95% CI=[0.02036, 0.5522]). In **Table 1** key indicators. The initial SI of the group C was significantly lower than that of the group PDN (*P* = 0.0368, 95% CI=[-5032, -101.4]). Calculating the spinal cord’s average hourly metabolism rate of the contrast agent (SIMR, see **Table 1** for the formula) further confirmed the impaired clearance efficiency and impaired glymphatic system function in PDN rats. The group OMT showed that OMT treatment effectively improved spinal cord contrast agent clearance (*P* = 0.0183, 95% CI=[-0.7526, -0.08606]), indicating an enhanced glymphatic system function. However, after PMA administration, the therapeutic effect of OMT was weakened (*P* = 0.0301, 95% CI=[-0.7089, -0.04235]), suggesting that PMA may block the mechanism of OMT and suppress its therapeutic effect, thereby hindering the recovery of glymphatic system function.

**Table 1:** This shows the detailed MRI SI values obtained from the lumbar enlargement.

	Group C (n=10)	Group PDN (n=10)	Group OMT (n=10)	Group PMA (n=10)	*P-value*
Initial	4172 ± 344.67	6738 ± 675.07*	4355.54 ± 396.68	5395 ± 500.18	0.0368
5-minute SI	4659 ± 223.65	8414 ± 1789.05***	4912 ± 225.305^##^	6727 ± 265.42	0.0006
PEAK SI	6025 ± 339.94	12347 ± 1541.10**	7685 ± 1138.17^##^	9779 ± 1374.68*	0.0011
SI Change	1853 ± 15.48	5608 ± 880.66*	3330 ± 1146.93	4384 ± 1873.29	0.0278
SIMR	0.40 ± 0.02	0.11 ± 0.01*	0.59 ± 0.01^#^	0.44 ± 0.18^#^	0.0353

The table displays the SI-related parameters: initial:the initial SI; 5-minute SI: SI at 5 minutes; peak SI:the peak SI; SI change: the change in SI from the initial value to the peak value; SIMR: the hourly metabolism rate of the spinal cord from 5 minutes to the peak time, calculated as SIMR=( Mean ( peak SI)-Mean ( initial value))/( Mean ( peak time)×Mean ( 5-minute SI )). Data are expressed as mean ± SD. * versus Group C; # versus Group PDN. *#, P<0.05; **##, P<0.01; ***, P<0.001.

### 3.3 Effects of OMT on oxidative stress and inflammatory factors in spinal cord tissue of PDN rats

**Figure 4:**
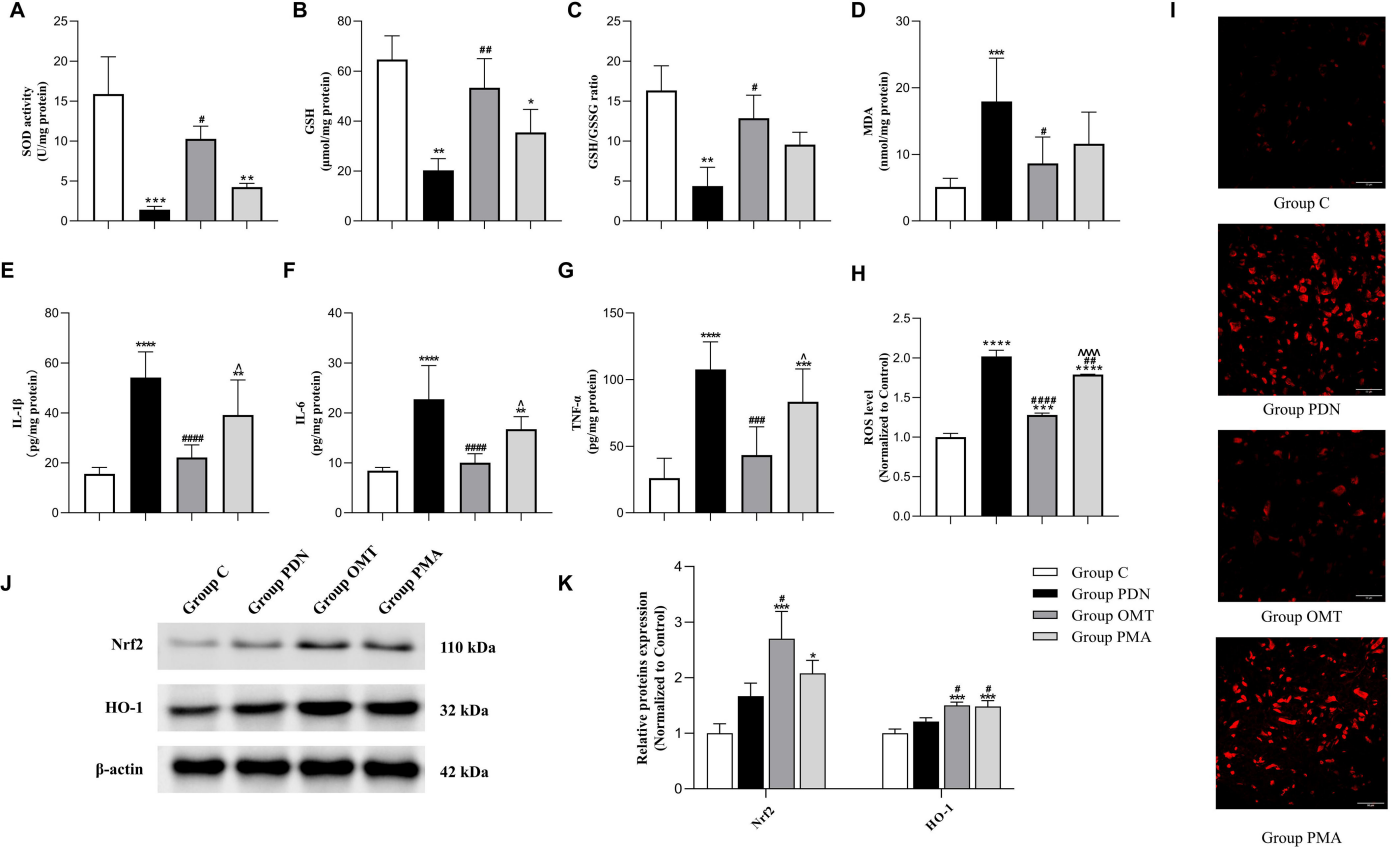
Detection results of oxidative stress and inflammatory factor-related indicators. **(A)** Detection results of SOD activity; **(B)** Detection results of GSH; **(C)** GSH/GSSG ratio; **(D)** MDA levels; **(E)** IL-1β levels; **(F)** IL-6 levels; **(G)** TNF-α levels; **(H)** ROS levels; **(I)** DHE probe staining of the lumbar enlargement of the spinal cord (×40); **(J)** Western blot bands of Nrf2 and HO-1; **(K)** Columnar chart of Nrf2 and HO-1. * versus Group C; # versus Group PDN; ∧ versus Group OMT. *#∧, P<0.05; **##, P<0.01; ***###, P<0.001; ****####∧∧∧∧, P<0.0001.

To explore the regulatory effects of OMT on OS and inflammatory factor levels in PDN rats, this experiment detected the expression levels of Nrf2 and its downstream antioxidant target protein HO-1 in spinal cord tissue, as well as indicators related to OS and inflammatory factors. As shown in **Figures 4A-D**, the OS status in the group PDN was significantly exacerbated. The activity of SOD (*P* = 0.0006, 95% CI=[7.385, 21.55]) and the level of GSH (*P* = 0.0015, 95% CI=[20.61, 68.18]) were significantly reduced compared with the group C, while the levels of MDA (*P* = 0.0005, 95% CI=[-20.48, -5.149]) and ROS (*P* < 0.001, 95% CI=[-1.152, -0.8837]) were significantly increased. Moreover, the WB results shown in **Figures 4J, K** indicated that the expression of Nrf2 and HO-1 was up-regulated in the group PDN, indicating that the Nrf2 signalling pathway was activated. The activation of the Nrf2 pathway can enhance the body’s antioxidant capacity and reduce the level of OS. However, the decrease in antioxidant indicators in the group PDN suggests that the OS has exceeded the compensatory capacity of the Nrf2 pathway, resulting in a severe imbalance of OS. Meanwhile, the results of inflammatory factor detection (**Figures 4E-G**) show that the levels of IL-1β, IL-6, and TNF-α in the spinal cord tissue of rats in the group PDN are significantly increased.

Compared with the group PDN, the group OMT exhibited significant improvements in the relevant OS indicators, and the levels of inflammatory factors were reduced. Moreover, in both the group OMT and PMA, the expression of Nrf2 and HO-1 was higher than that in the group PDN, indicating that OMT can further enhance the antioxidant capacity of rats via the Nrf2 pathway and effectively improve the OS status and inflammatory response in PDN rats. In contrast, although there was a certain degree of reduction in OS levels in the group PMA, it did not reach the significant level of the group OMT, and the levels of inflammatory factors were not significantly reduced (all P>0.05). This indicates that PMA, by activating the NF-κB signalling pathway, affected the therapeutic effect of OMT.

### 3.4 Hyperactivation of the NF-κB pathway and upregulation of MMP-9

**Figure 5:**
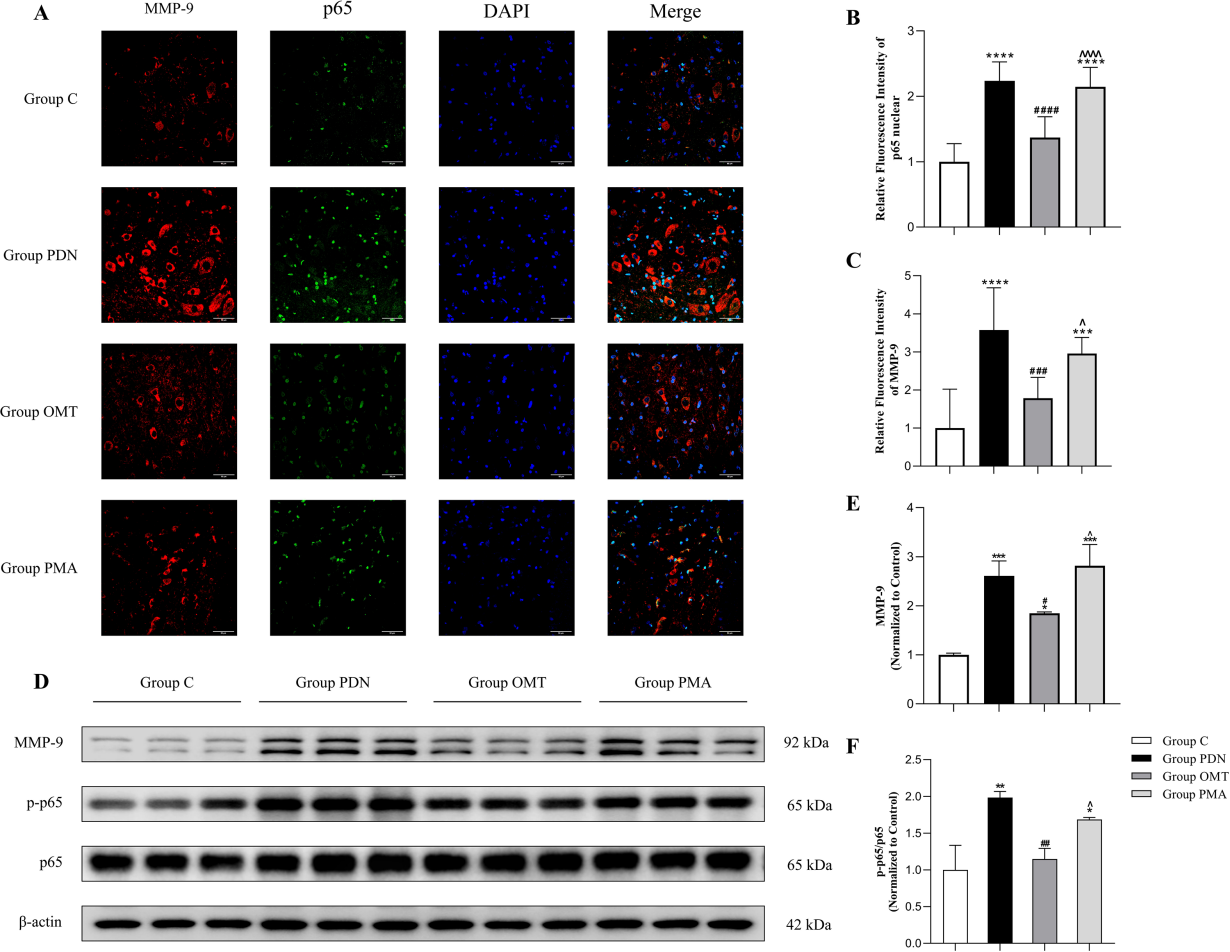
Detection Results of NF-κB p65 and MMP-9. **(A)** Immunofluorescence staining of the lumbar enlargement of the spinal cord in rats. p65 was labelled with green fluorescence, MMP-9 was labelled with red fluorescence, and nuclei were counterstained with DAPI (blue). The slices show the nuclear translocation rate of p65 and the expression of MMP-9; **(B)** Analysis of the fluorescence intensity of p65 in the nucleus relative to the C group; **(C)** Analysis of the fluorescence intensity of MMP-9 relative to the C group; **(D)** Western blot bands of MMP-9, NF-κB p65 and p-p65 in four spinal groups; **(E)** Columnar chart of MMP-9; **(F)** columnar chart of p-p65/p65 ratio; * versus Group C; # versus Group PDN; ∧ versus Group OMT. *#∧, P<0.05; **##, P<0.01; ***###, P<0.001; ****####∧∧∧∧, P<0.0001.

To assess the activation of the NF-κB pathway and MMP-9 expression in each group, we sectioned the lumbar enlargement of the spinal cord. NF-κB p65 nuclear expression and MMP-9 were detected by immunofluorescence. The p-p65/p65 ratio was calculated and MMP-9 expression was measured by Western blot for further validation. In **Figures 5A-C**, in the group PDN, NF-κB p65 nuclear expression rose significantly (*P* < 0.001, 95% CI=[-1.628, -0.8462]), indicated by stronger nuclear green fluorescence, suggesting overactivation of the NF-κB pathway. Meanwhile, MMP-9 expression (red fluorescence) increased markedly versus the control group (*P* < 0.001, 95% CI=[-3.674, -1.472]). OMT treatment significantly inhibited the nuclear translocation of NF-κB p65 (*P* < 0.001, 95% CI=[0.4759, 1.258]), yet its nuclear expression remained higher than that of group C, and the fluorescence intensity of MMP-9 showed a similar trend to the activation degree of the NF-κB signalling pathway. In the group PMA, the nuclear expression level of NF-κB p65 was significantly increased (*P* < 0.001, 95% CI=[-1.167, -0.3852]), with a concurrent upward trend in the fluorescence intensity of MMP-9 (*P* = 0.0317, 95% CI=[-2.273, -0.07074]). In **Figures 5D-F**, consistent with the immunofluorescence findings, the phosphorylation level of p65 in the spinal cord tissue of the group PDN was significantly higher than that of the group C (*P* = 0.0012, 95% CI=[-1.517, -0.4533]), with a corresponding significant increase in the expression level of MMP-9 *(P* = 0.0004, 95% CI=[-2.363, -0.8546]). After OMT treatment, the phosphorylation of p65 and the expression of MMP-9 were both reduced, whereas no significant improvement was observed in the group PMA. Thus, the NF-κB signalling pathway may be associated with the regulation of MMP-9 expression, and OMT exerts its therapeutic effects by downregulating MMP-9 expression through the inhibition of NF-κB nuclear translocation.

### 3.5 Astrocyte activation analysis and AQP4 polarity changes

**Figure 6:**
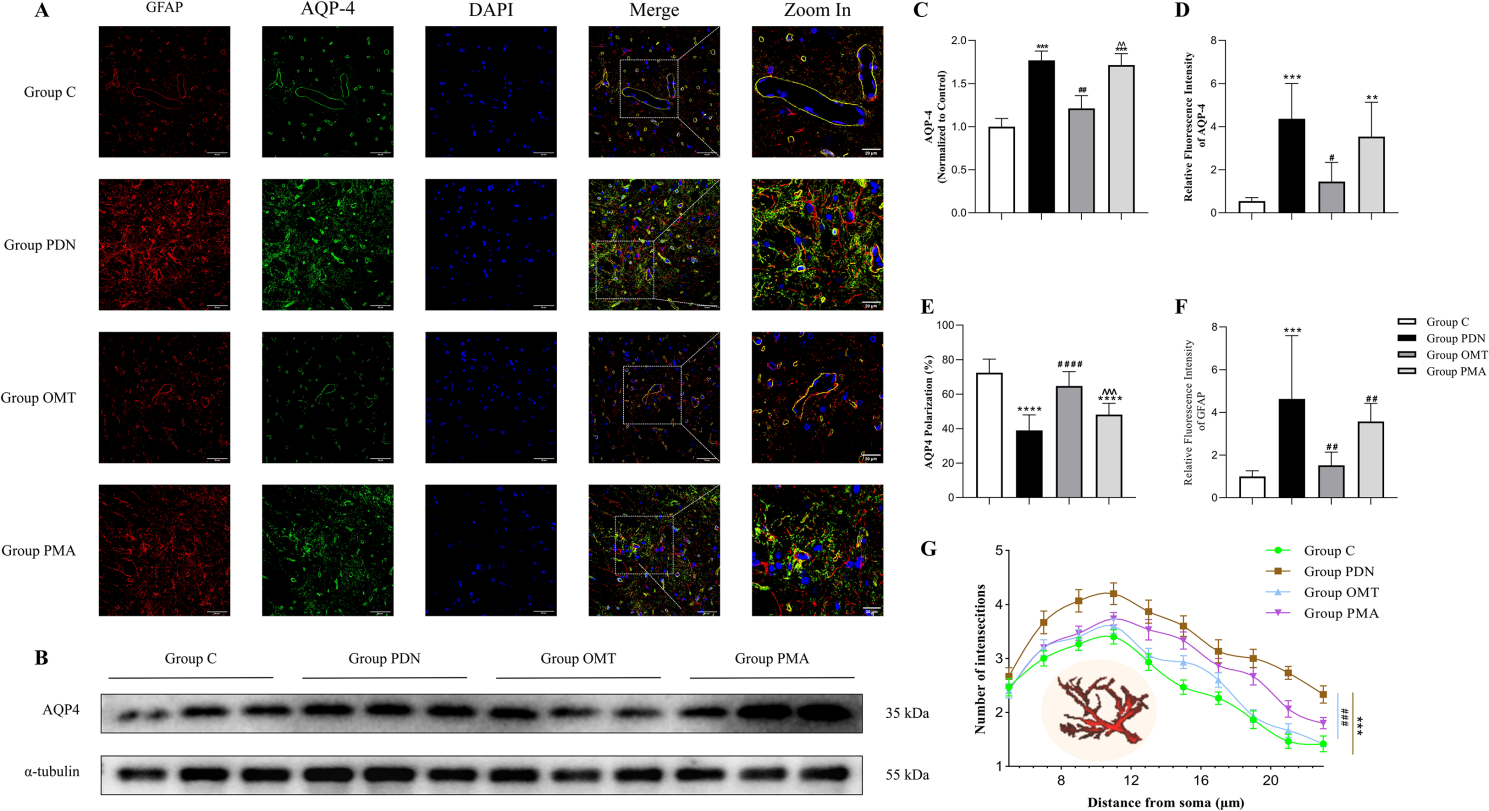
Immunofluorescence of AQP-4 and GFAP and Western Blot of AQP-4. **(A)** Immunofluorescence staining of spinal cord sections. AQP-4 was labelled with green fluorescence, GFAP was labelled with red fluorescence, and nuclei were counterstained with DAPI (blue). The figure shows the changes in AQP-4 polarity. Scale bar=50µm. **(B)** Western blot bands of AQP4; **(C)** columnar chart of AQP4 expression; **(D)** Quantitative analysis of AQP-4 fluorescence intensity relative to group C; **(E)** Comparative analysis of AQP-4 polarity rate. **(F)** Quantitative analysis of GFAP fluorescence intensity relative to group C; **(G)** Sholl analysis of astrocyte images. * versus Group C; # versus Group PDN; ∧ versus Group OMT; #, P<0.05; ##∧∧, P<0.01; ***###∧∧∧, P<0.001; ****####, P<0.0001.

The proper polarization of AQP4 is crucial for glymphatic system function. In this study, immunofluorescence was used to evaluate AQP4 polarization in spinal cord astrocytes across rat groups, and WB was used to measure AQP4 expression levels. GFAP, marked by red fluorescence, served as a specific astrocyte marker. AQP4 expression and polarization changes at the astrocyte end-feet were indicated by green fluorescence. In **Figure 6A**, the group C exhibited typical AQP4 polarization around blood vessels within astrocyte end-feet, marked by red GFAP fluorescence. In contrast, the group PDN showed increased and dispersed AQP4 expression with irregular localization, indicating depolarization (C: 72.41 ± 7.92; PDN: 38.97 ± 9.00, *P* < 0.001, 95% CI=[22.82, 44.06]). AQP4 polarization was calculated from three images per section using the formula, with the average value representing the sample’s AQP4 polarization rate. Compared to the group PDN, the group OMT showed significant improvement in AQP4 polarization (64.74 ± 8.33, *P* < 0.001, 95% CI=[-36.39, -15.15]), while the group PMA showed no significant difference (48.10 ± 6.59, *P* = 0.1286, 95% CI=[-19.75, 1.485]). These results indicate that OMT can restore AQP4 polarization to exert a regulatory effect, while PMA interferes with the therapeutic effect of OMT by activating the NF-κB signalling pathway.

We next analysed the morphological complexity and fluorescence intensity of astrocytes. In **Figures 6F, G**, astrocytes in the group PDN exhibited more complex morphology and significantly higher overall fluorescence intensity. This phenomenon indicates that the activation state of astrocytes in PDN rats is enhanced, which may be to regulate the inflammatory response and reduce the damage of inflammation to neurons. Meanwhile, due to impaired AQP4 polarization leading to dysfunction of the glymphatic system, astrocytes compensatorily increase the complexity and number of their processes, enhancing connections with blood vessels to promote the clearance of inflammatory mediators and the supply of nutrients. After OMT treatment, the activation state of astrocytes was reduced, but this effect was blocked by PMA. Finally, AQP4 expression levels were analysed by immunofluorescence and WB, with consistent results showing elevated AQP4 expression in the group PDN. OMT could reduce this overexpression, bringing it closer to the levels seen in normal rats.

### 3.6 Results of network pharmacology analysis

**Figure 7:**
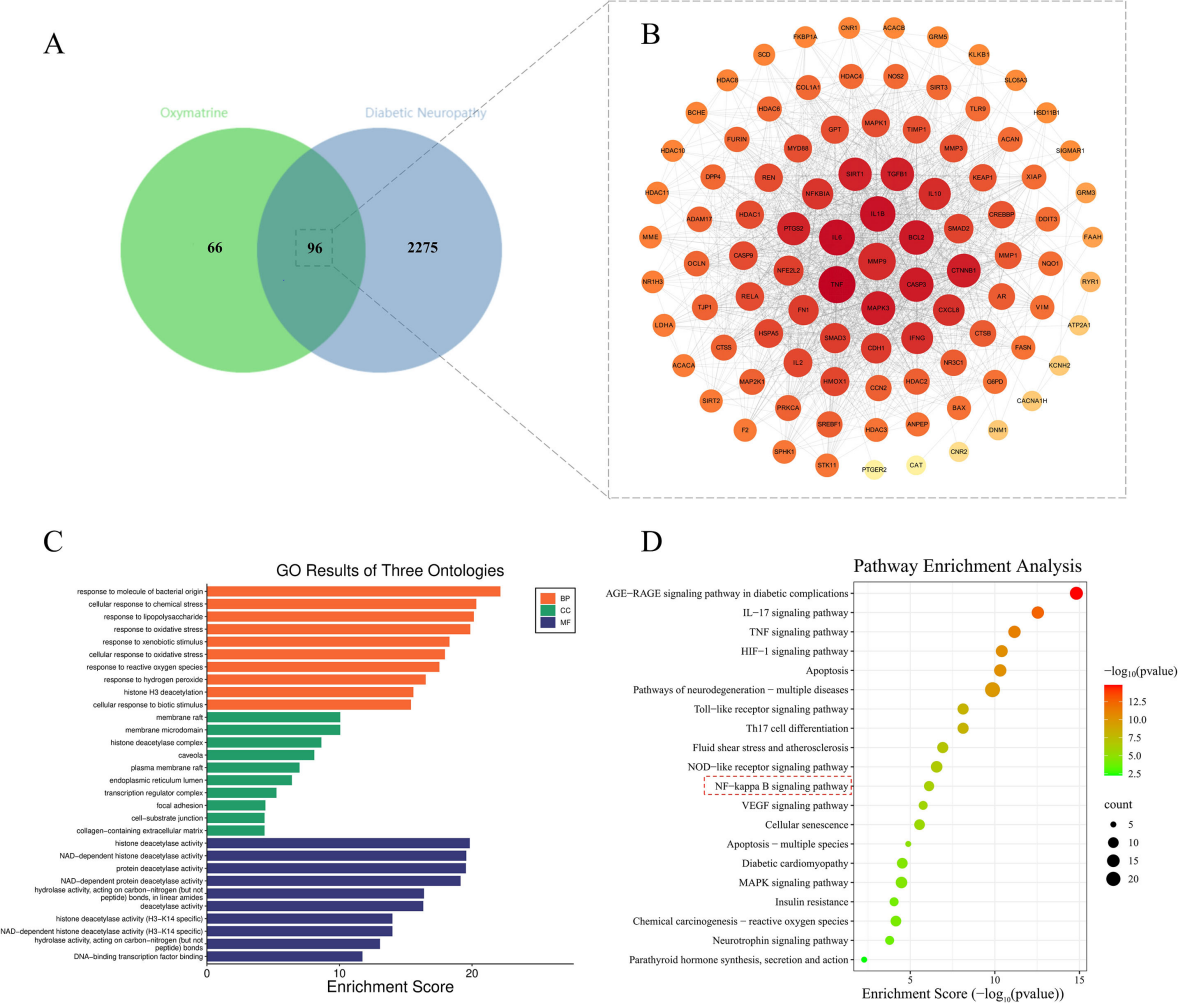
Targets and functional analysis of OMT in intervening diabetic neuropathy. **(A)** The Venn diagram shows the common target genes of OMT and diabetic neuropathy (a total of 96). **(B)** The PPI network presents the protein-protein interaction relationships among key targets. **(C)** The GO enrichment analysis plot displays the classification of biological functions involved in the targets. BP stands for biological process, CC for cellular component, and MF for molecular function. **(D)** The KEGG enrichment bubble plot shows the related signalling pathways involved in the targets.

As shown in **Figure 7**, network pharmacology analysis identified 96 intersection targets between OMT and diabetic neuropathy, among which MMP9 is one of the core targets. Enrichment analysis revealed that these targets were significantly enriched in the NF-κB signalling pathway and OS regulatory pathway. This result suggested that OMT may inhibit the inflammatory response by regulating the NF-κB pathway, while enhancing the Nrf2 pathway to regulate OS and alleviate damage, and can target MMP9 to improve the polarized localization of AQP4. This was completely consistent with the pharmacological effects of OMT observed in *in vivo* experiments, providing hypothetical support for OMT to alleviate PDN by regulating the aforementioned pathways and targets, and also offering a theoretical basis for OMT as a therapeutic option for PDN.

## 4 Discussion

In this study, we investigated whether OMT could improve spinal cord OS and inflammatory factors in PDN rats, and whether it could restore glymphatic function and alleviate PDN symptoms. The results showed that PDN rats exhibited increased OS and elevated levels of inflammatory factors, with impaired spinal cord glymphatic function, which may be related to ROS and associated inflammatory factors (48). Elevated OS and inflammation can directly damage the glymphatic system and activate the NF-κB pathway, creating a vicious cycle (49). NF-κB activation also upregulates MMP-9, further disrupting AQP4 polarization (50). Additionally, the overactivation of astrocytes may also trigger neuroinflammation, metabolic disorders, and dysfunction of the neural network (51). Together, these mechanisms cause glymphatic system dysfunction and promote PDN development.

OMT treatment enhanced the antioxidant capacity of PDN rats, reduced OS levels, and decreased the production of inflammatory factors. PMA, by activating the NF-κB signalling pathway, attenuated the therapeutic effects of OMT, resulting in less significant improvements in OS and inflammatory factors compared to the group OMT. These findings suggest a potential interplay between inflammatory factors and OS, with OMT modulating both the Nrf2 and NF-κB pathways to synergistically ameliorate OS and inflammatory responses, thereby alleviating PDN symptoms, and finally, network analysis further provides hypothetical support for this mechanism.

To determine whether PDN rats have glymphatic system dysfunction and whether OMT can restore it, this study used MRI to measure SI changes in the spinal cord’s lumbar enlargement region after contrast agent injection (52). Sevoflurane was used for rat anaesthesia to minimize effects on the glymphatic system. Results showed that PDN rats had significantly higher baseline and peak SI than the control group. This might be due to long-term glymphatic dysfunction causing metabolic waste and inflammatory factor accumulation (53) or AQP4 overexpression leading to spinal cord oedema after polarity loss (54). In addition, by calculating the average hourly metabolic rate of the contrast agent in rats of each group, this study further verified that PDN rats have definite glymphatic system dysfunction, and their metabolic waste clearance efficiency is significantly reduced. Moreover, the continuous accumulation of metabolic waste will further inhibit the metabolic transport rate of the glymphatic system through mechanisms such as disturbing the interstitial fluid concentration gradient and damaging vascular endothelial function (55), eventually forming a vicious circle and aggravating the pathological process of PDN. Due to MRI resolution limitations, we couldn’t precisely observe metabolic waste clearance pathways and speeds in the spinal cord, restricting our understanding of glymphatic clearance. Future studies could employ a variety of technical approaches to improve this. For example, specific fluorescent probes could be used to label metabolic products and vascular structures, and then combined with Two-Photon Microscopy to track the clearance pathways and dynamics of metabolic waste in real time (56), thereby obtaining more detailed information on the clearance process. Alternatively, a laser speckle system could be used to observe blood flow perfusion and the degree of vasodilation in the spinal cord vessels (57), thereby providing a comprehensive assessment of vascular function. This technology can offer high-resolution images of blood flow perfusion, which may help reveal the relationship between vascular function and glymphatic system function.

In the glymphatic system, proper AQP4 polarization promotes rapid CSF-ISF exchange, enhancing metabolic waste clearance (58). In this study, it was observed that the expression level of AQP4 tends to increase in PDN rats. This finding is consistent with some previous studies (59), suggesting that under specific pathological conditions, such as neuroinflammation or tissue damage, the expression of AQP4 is upregulated (60). As a core molecule regulating the function of the glymphatic system, the upregulated expression of AQP4 in the early stage of disease can enhance transmembrane water transport, and alleviate the compressive injury of local tissue oedema to nerve fibres (61); it can also accelerate the excretion of toxic metabolites in nerve tissue by enhancing the fluid circulation efficiency of the glymphatic system (62), thereby reducing the damage of their accumulation to neurons and myelin sheaths. This upregulation is a compensatory mechanism of the body, which aims to enhance fluid mobility and metabolic waste clearance capacity to meet the physiological needs under pathological conditions. However, some studies have drawn opposite conclusions. These discrepancies may involve multiple aspects. First, there are differences in experimental design. The PDN animal models selected in different studies are inconsistent (63); for example, the models induced by a single high-dose STZ injection and multiple low-dose STZ injections show significant differences in the duration of hyperglycaemia exposure, the progression rate of neuroinflammation, and the degree of glial cell activation, thereby affecting the regulatory effect on AQP4 expression. Second, there are stage-specific differences in the disease process (64). In the advanced stage of the disease, under continuous oxidative stress and inflammatory stimulation, the expression of AQP4 may show a downward trend. Finally, there is the bidirectional expression characteristic of AQP4 in central nervous system diseases (65). The expression of AQP4 is subject to the cross-regulation of multiple signalling pathways (66, 67), and it can exhibit completely opposite trends under the action of different inflammatory factors and microenvironments, which further exacerbates the discrepancies among research conclusions. Therefore, the function of AQP4 in the CNS exhibits a high degree of complexity, and its expression levels are regulated by multiple factors (68). In future research, we can further explore the reasons for these differing results. This could help clarify the pathological role of AQP4 in CNS diseases and provide a more accurate theoretical basis for the treatment of related diseases.

Based on the previous results, astrocytes in PDN rats are significantly activated. We speculate that this activation is a protective mechanism initiated by the body under pathological conditions to inhibit excessive inflammatory responses and protect neural tissue from inflammatory damage (69), with the aim of creating a favourable microenvironment for nerve repair. Moreover, the activated astrocytes extend more foot processes to closely envelop the central vessel (70), becoming a key component of the glymphatic system, thereby enhancing the exchange between CSF and ISF. This is consistent with the purpose of upregulated AQP4 expression, both aiming to enhance the metabolic function of the spinal cord glymphatic system. However, excessive activation of astrocytes may also trigger neuroinflammation, metabolic disturbances, and dysfunction of neural networks (71). When A1-type reactive astrocytes proliferate excessively, they can not only induce central sensitization, hyperalgesia, and maintenance of chronic pain by secreting pro-inflammatory factors and neurotoxins (72), but also regulate the release of neurotransmitters (73), interfere with the balance of emotion-related brain regions, and induce pain-depression comorbidity. Similarly, when AQP4 is overexpressed, its excessive aggregation in the cell body or membrane can promote the formation of oedema and ion disturbances (27), thereby exacerbating neuroinflammation. Therefore, it is not the changes in AQP4 expression levels that are key, but rather the alterations in its polarization state that are the critical factors for the dysfunction of the glymphatic system. The correct polarization of AQP4 is influenced by a variety of factors. Previous studies have shown that MMP-9 can disrupt AQP4 polarization by cleaving β-DG (74), and that glymphatic dysfunction severity correlates positively with MMP-9 levels. Consistent with this, our study found significantly elevated MMP-9 expression in PDN rats. Compared with the improvement in the group OMT, the expression of MMP9 in the group PMA was still relatively high, accompanied by glymphatic system dysfunction. As confirmed in our previous study, NLRP3 can affect the polarized localization of AQP4 by regulating the expression of MMP9 (40); thus, the activation of the NF-κB pathway may exert its effect through this mechanism. In addition, excessive accumulation of MMP9 can degrade tight junction proteins, thereby damaging the integrity of the blood-spinal cord barrier (75). This will lead to the invasion of peripheral immune cells into the central nervous system, aggravate neuroinflammation, and induce the occurrence of pain. Previous studies have predominantly focused on the role of the NF-κB pathway in the generation of inflammatory factors and neuroinflammation, as well as the regulation of AQP4 expression levels by inflammatory factors. This study initially clarifies the specific mechanisms by which the high OS and elevated inflammatory factor levels caused by hyperglycaemia in diabetes regulate the expression of MMP9 through the NF-κB pathway, thereby affecting the polarization state of AQP4 and leading to dysfunction of the glymphatic system. This finding not only provides a new perspective for a deeper understanding of the roles of OS and inflammation in the pathogenesis of PDN, but also offers a theoretical basis for the potential application value of OMT in the treatment of PDN.

Although this study has made important discoveries, there are still some limitations. First, the exact molecular mechanisms of NF-κB pathway regulation of MMP-9 expression and its impact on AQP4 polarization are not fully understood. Future research needs to further explore the precise molecular mechanisms between the NF-κB pathway and AQP4 polarization to better understand the mechanisms of glymphatic system dysfunction and provide a stronger theoretical basis for treating PDN and other CNS diseases. In addition, the method for assessing AQP4 polarization in this study is relatively macroscopic and lacks detailed analysis of cellular morphological changes. Future research could combine electron microscopy to precisely evaluate AQP4 polarization at the cellular level (76), which would deepen our understanding of the recovery mechanisms of glymphatic system function. While the antioxidant effect of OMT in this study has been preliminarily supported by the changes in ROS, MDA, SOD, and GSH levels, and the activation of the Nrf2 pathway has been evaluated by the expression of Nrf2 and HO-1, the study has not yet involved the nuclear translocation or nuclear localization of Nrf2. Considering that the nuclear translocation of Nrf2 is an important criterion for its activation state, future studies will more accurately assess the ability of OMT to respond to oxidative stress via the Nrf2 pathway by evaluating the nuclear translocation of Nrf2. Even though PMA can block the therapeutic effect of OMT, OMT may still exert its effects through other mechanisms, which makes the inference of causal relationship not sufficiently direct. To more clearly verify the specific role of the NF-κB pathway in PDN rats, future studies may consider adding an experimental group treated with a specific NF-κB inhibitor to further refine the research findings. Additionally, because this study did not detect the insulin levels in diabetic rats and only used a single behavioural index to evaluate pain, it failed to comprehensively assess their insulin resistance status and the various characteristics of neuropathic pain. Future studies will supplement the detection of insulin levels and insulin resistance-related indicators, and at the same time increase multimodal pain assessment methods such as hot plate test and spontaneous pain scoring to more accurately evaluate the PDN model and intervention effects. A further limitation is that the small sample size in some experiments, the significance of the results was affected. Future research should increase the sample size to enhance the credibility of the results. Finally, the efficacy of OMT at different disease stages and its synergistic effects with other treatments can be further investigated. In the future, we plan to further explore the specific molecular mechanisms of OMT’s antioxidant effects and conduct a comprehensive analysis of its mechanisms of action.

In summary, this study has thoroughly investigated the mechanisms by which OMT ameliorates the function of the glymphatic system by improving the spinal cord OS status and levels of inflammatory factors in PDN rats. The results indicate that OMT significantly enhances the antioxidant capacity in PDN rats and effectively reduces OS levels by activating the Nrf2 pathway. Meanwhile, OMT also reduces the production of inflammatory factors and regulates the expression of MMP-9 by inhibiting the NF-κB pathway. These changes collectively improve the activation state of astrocytes, restore the polarized localization of AQP4, and enhance the function of the glymphatic system, thereby effectively alleviating PDN symptoms. This finding provides a substantial theoretical basis for the potential application of OMT in PDN treatment.

## Data Availability

The original contributions presented in the study are included in the article/Supplementary Material. Further inquiries can be directed to the corresponding author.

## References

[1] Ong KL Stafford LK McLaughlin SA Boyko EJ Vollset SE Smith AE . Global, regional, and national burden of diabetes from 1990 to 2021, with projections of prevalence to 2050: a systematic analysis for the Global Burden of Disease Study 2021. Lancet. (2023) 402:203–34. doi: 10.1016/S0140-6736(23)01301-6, PMID: 37356446 PMC10364581

[2] Chen M Dou C Ye C Kong L Xu M Xu Y . Socioeconomic and mental health inequalities in global burden of type 2 diabetes: Evidence from the Global Burden of Disease Study 2021. Diabetes Obes Metab. (2025) 27:1792–804. doi: 10.1111/dom.16173, PMID: 39763005

[3] Abbott CA Malik RA Van Ross ERE Kulkarni J Boulton AJM . Prevalence and characteristics of painful diabetic neuropathy in a large community-based diabetic population in the U.K. Diabetes Care. (2011) 34:2220–4. doi: 10.2337/dc11-1108, PMID: 21852677 PMC3177727

[4] Scholz J Rathmell JP David WS Chad DA Broderick AC Perros SG . A standardized clinical evaluation of phenotypic diversity in diabetic polyneuropathy. Pain. (2016) 157:2297–308. doi: 10.1097/j.pain.0000000000000648, PMID: 27322439

[5] Gylfadottir SS Christensen DH Nicolaisen SK Andersen H Callaghan C Itani M . Diabetic polyneuropathy and pain, prevalence, and patient characteristics: A cross- sectional questionnaire study of 5,514 patients with early type 2 diabetes. PloS One. (2022) 161(3):574–83. doi: 10.1097/j.pain.0000000000001744, PMID: 31693539 PMC7017941

[6] Tesfaye S Vileikyte L Rayman G Sindrup SH Perkins BA Baconja M . Painful diabetic peripheral neuropathy: consensus recommendations on diagnosis, assessment and management. Diabetes Metab Res. (2011) 27:629–38. doi: 10.1002/dmrr.1225, PMID: 21695762

[7] Griebeler ML Tsapas A Brito JP Wang Z Phung OJ Montori VM . Pharmacologic interventions for painful diabetic neuropathy: an umbrella systematic review and comparative effectiveness network meta-analysis (Protocol). Syst Rev. (2012) 1:61. doi: 10.1186/2046-4053-1-61, PMID: 23198755 PMC3534585

[8] Mallick-Searle T Adler J . Update on treating painful diabetic peripheral neuropathy: A review of current US guidelines with a focus on the most recently approved management options. JPR. (2024) 17:1005–28. doi: 10.2147/JPR.S442595, PMID: 38505500 PMC10949339

[9] Jessen NA Munk ASF Lundgaard I Nedergaard M . The glymphatic system: A beginner’s guide. Neurochem Res. (2015) 40:2583–99. doi: 10.1007/s11064-015-1581-6, PMID: 25947369 PMC4636982

[10] Iliff JJ Wang M Zeppenfeld DM Venkataraman A Plog BA Liao Y . Cerebral arterial pulsation drives paravascular CSF–interstitial fluid exchange in the murine brain. J Neurosci. (2013) 33:18190–9. doi: 10.1523/JNEUROSCI.1592-13.2013, PMID: 24227727 PMC3866416

[11] Liu S Lam MA Sial A Hemley SJ Bilston LE Stoodley MA . Fluid outflow in the rat spinal cord: the role of perivascular and paravascular pathways. Fluids Barriers CNS. (2018) 15:13. doi: 10.1186/s12987-018-0098-1, PMID: 29704892 PMC5924677

[12] Carlstrom LP Eltanahy A Perry A Rabinstein AA Elder BD Morris JM . A clinical primer for the glymphatic system. Brain. (2022) 145:843–57. doi: 10.1093/brain/awab428, PMID: 34888633

[13] Lan Y Wang H Chen A Zhang J . Update on the current knowledge of lymphatic drainage system and its emerging roles in glioma management. Immunology. (2023) 168:233–47. doi: 10.1111/imm.13517, PMID: 35719015

[14] Gao Y Liu K Zhu J . Glymphatic system: an emerging therapeutic approach for neurological disorders. Front Mol Neurosci. (2023) 16:1138769. doi: 10.3389/fnmol.2023.1138769, PMID: 37485040 PMC10359151

[15] Mestre H Hablitz LM Xavier ALR Feng W Zou W Pu T . Aquaporin-4-dependent glymphatic solute transport in the rodent brain. eLife. (2018) 7:e40070. doi: 10.7554/eLife.40070, PMID: 30561329 PMC6307855

[16] Park H Choi S-H Kong M-J Kang T-C . Dysfunction of 67-kDa Laminin Receptor Disrupts BBB Integrity via Impaired Dystrophin/AQP4 Complex and p38 MAPK/VEGF Activation Following Status Epilepticus. Front Cell Neurosci. (2019) 13:236. doi: 10.3389/fncel.2019.00236, PMID: 31178701 PMC6542995

[17] Bhat HF Mir SS Dar KB Bhat ZF Shah RA Ganai NA . ABC of multifaceted dystrophin glycoprotein complex (DGC). J Cell Physiol. (2018) 233:5142–59. doi: 10.1002/jcp.25982, PMID: 28464259

[18] Dossena S Marino A . Cellular oxidative stress. Antioxidants. (2021) 10:399. doi: 10.3390/antiox10030399, PMID: 33800761 PMC7998990

[19] Chen QM . Nrf2 for cardiac protection: pharmacological options against oxidative stress. Trends Pharmacol Sci. (2021) 42:729–44. doi: 10.1016/j.tips.2021.06.005, PMID: 34332753 PMC8785681

[20] Canning P Sorrell FJ Bullock AN . Structural basis of Keap1 interactions with Nrf2. Free Radical Biol Med. (2015) 88:101–7. doi: 10.1016/j.freeradbiomed.2015.05.034, PMID: 26057936 PMC4668279

[21] Ngo V Karunatilleke NC Brickenden A Choy W-Y Duennwald ML . Oxidative stress-induced misfolding and inclusion formation of Nrf2 and Keap1. Antioxidants. (2022) 11:243. doi: 10.3390/antiox11020243, PMID: 35204126 PMC8868093

[22] Duan C Wang H Jiao D Geng Y Wu Q Yan H . Curcumin restrains oxidative stress of after intracerebral hemorrhage in rat by activating the Nrf2/HO-1 pathway. Front Pharmacol. (2022) 13:889226. doi: 10.3389/fphar.2022.889226, PMID: 35571134 PMC9092178

[23] Sies H Berndt C Jones DP . Oxidative stress. Annu Rev Biochem. (2017) 86:715–48. doi: 10.1146/annurev-biochem-061516-045037, PMID: 28441057

[24] Arı M Erdogan MA Erbaş O . Investigation of the protective effects of dichloroacetic acid in a rat model of diabetic neuropathy. BMC Pharmacol Toxicol. (2025) 26:15. doi: 10.1186/s40360-025-00849-8, PMID: 39844306 PMC11756200

[25] Román-Pintos LM Villegas-Rivera G Rodríguez-Carrizalez AD Miranda-Díaz AG Cardona-Muñoz EG . Diabetic polyneuropathy in type 2 diabetes mellitus: inflammation, oxidative stress, and mitochondrial function. J Diabetes Res. (2016) 2016:1–16. doi: 10.1155/2016/3425617, PMID: 28058263 PMC5183791

[26] Sousa NA Oliveira GAL de Oliveira AP Lopes ALF Iles B Nogueira KM . Novel ocellatin peptides mitigate LPS-induced ROS formation and NF-kB activation in microglia and hippocampal neurons. Sci Rep. (2020) 10:2696. doi: 10.1038/s41598-020-59665-1, PMID: 32060388 PMC7021831

[27] Lu H Ai L Zhang B . TNF-α induces AQP4 overexpression in astrocytes through the NF-κB pathway causing cellular edema and apoptosis. Bioscience. (2022) 42:BSR20212224. doi: 10.1042/BSR20212224, PMID: 35260880 PMC8935387

[28] Bi C Tham DKL Perronnet C Joshi B Nabi IR Moukhles H . The oxidative stress-induced increase in the membrane expression of the water-permeable channel aquaporin-4 in astrocytes is regulated by caveolin-1 phosphorylation. Front Cell Neurosci. (2017) 11:412. doi: 10.3389/fncel.2017.00412, PMID: 29326556 PMC5742350

[29] Li Y Zhang L Zhang Y Miao Z Liu Z Zhou G . Potential molecular mechanism of Guiqi Baizhu Decoction in radiation-induced intestinal edema by regulating HIF-1a, AQP4 and Na+/K+-ATPase. Phytomedicine. (2022) 107:154445. doi: 10.1016/j.phymed.2022.154445, PMID: 36130463

[30] Yue Y Zhang X Lv W Lai H-Y Shen T . Interplay between the glymphatic system and neurotoxic proteins in Parkinson’s disease and related disorders: current knowledge and future directions. Neural Regen Res. (2024) 19:1973–80. doi: 10.4103/1673-5374.390970, PMID: 38227524 PMC11040291

[31] Ringland C Schweig JE Eisenbaum M Paris D Ait-Ghezala G Mullan M . MMP9 modulation improves specific neurobehavioral deficits in a mouse model of Alzheimer’s disease. BMC Neurosci. (2021) 22:39. doi: 10.1186/s12868-021-00643-2, PMID: 34034683 PMC8152085

[32] Tang J Yue J Tao Y Zhao G Yi X Zhang M . Neutrophil extracellular traps induce brain edema around intracerebral hematoma via ERK-mediated regulation of MMP9 and AQP4. Transl Stroke Res. (2024) 16(5):1461–73. doi: 10.1007/s12975-024-01318-w, PMID: 39733198 PMC12391242

[33] Si X Dai S Fang Y Tang J Wang Z Li Y . Matrix metalloproteinase-9 inhibition prevents aquaporin-4 depolarization-mediated glymphatic dysfunction in Parkinson’s disease. J Advanced Res. (2024) 56:125–36. doi: 10.1016/j.jare.2023.03.004, PMID: 36940850 PMC10834796

[34] Gu S Li Y Jiang Y Huang JH Wang F . Glymphatic dysfunction induced oxidative stress and neuro-inflammation in major depression disorders. Antioxidants. (2022) 11:2296. doi: 10.3390/antiox11112296, PMID: 36421482 PMC9687220

[35] Huan DQ Hop NQ Son NT . Oxymatrine: A current overview of its health benefits. Fitoterapia. (2023) 168:105565. doi: 10.1016/j.fitote.2023.105565, PMID: 37295753

[36] Hu Z-X Zhang J Zhang T Tian C-Y An Q Yi P . Aloperine-type alkaloids with antiviral and antifungal activities from the seeds of *Sophora alopecuroides* L. J Agric Food Chem. (2024) 72:8225–36. doi: 10.1021/acs.jafc.4c00992, PMID: 38557068

[37] An N Zhang G Li Y Yuan C Yang F Zhang L . Promising antioxidative effect of berberine in cardiovascular diseases. Front Pharmacol. (2022) 13:865353. doi: 10.3389/fphar.2022.865353, PMID: 35321323 PMC8936808

[38] Jiang Y Sang W Wang C Lu H Zhang T Wang Z . Oxymatrine exerts protective effects on osteoarthritis via modulating chondrocyte homoeostasis and suppressing osteoclastogenesis. J Cell Mol Medi. (2018) 22:3941–54. doi: 10.1111/jcmm.13674, PMID: 29799160 PMC6050479

[39] Southam K De Sousa C Daniel A Taylor BV Foa L Premilovac D . Development and characterisation of a rat model that exhibits both metabolic dysfunction and neurodegeneration seen in type 2 diabetes. J Physiol. (2022) 600:1611–30. doi: 10.1113/JP282454, PMID: 35128667 PMC9541365

[40] Jia S-Y Yin W-Q Xu W-M Li J Yan W Lin J-Y . Liquiritin ameliorates painful diabetic neuropathy in SD rats by inhibiting NLRP3-MMP- 9-mediated reversal of aquaporin-4 polarity in the glymphatic system. Front Pharmacol. (2024) 15:1436146. doi: 10.3389/fphar.2024.1436146, PMID: 39295943 PMC11408323

[41] Zhao Y Wang Q-Y Zeng L-T Wang J-J Liu Z Fan G-Q . Long-term high-fat high-fructose diet induces type 2 diabetes in rats through oxidative stress. Nutrients. (2022) 14:2181. doi: 10.3390/nu14112181, PMID: 35683981 PMC9182436

[42] Bonin RP Bories C De Koninck Y . A simplified up-down method (SUDO) for measuring mechanical nociception in rodents using von Frey filaments. Mol Pain. (2014) 10:1744–8069–10–26. doi: 10.1186/1744-8069-10-26, PMID: 24739328 PMC4020614

[43] De Sousa MVP Ferraresi C De Magalhães AC Yoshimura EM Hamblin MR . Building, testing and validating a set of home-made von Frey filaments: A precise, accurate and cost effective alternative for nociception assessment. J Neurosci Methods. (2014) 232:1–5. doi: 10.1016/j.jneumeth.2014.04.017, PMID: 24793398 PMC4136637

[44] Hu X Jiang J Leng Y Yang Y Zhang D Li K . Characterization of neuronal spiking patterns in the medial prefrontal cortex under varied general anesthetics in mice. Anesthesiol Perioper Sci. (2025) 3:10. doi: 10.1007/s44254-025-00092-8

[45] Lin J Li X Yang Y Ge Z Liu D Yang C . Protective effects of sevoflurane conditioning against myocardial ischemia-reperfusion injury: a review of evidence from animal and clinical studies. Anesthesiol Perioper Sci. (2025) 3:4. doi: 10.1007/s44254-024-00084-0

[46] Wang J Deng X . Inadvertent hypothermia: a prevalent perioperative issue that remains to be improved. Anesthesiol Perioper Sci. (2023) 1:24. doi: 10.1007/s44254-023-00022-6

[47] Ferreira TA Blackman AV Oyrer J Jayabal S Chung AJ Watt AJ . Neuronal morphometry directly from bitmap images. Nat Methods. (2014) 11:982–4. doi: 10.1038/nmeth.3125, PMID: 25264773 PMC5271921

[48] Xiong Y Yu Q Zhi H Peng H Xie M Li R . Advances in the study of the glymphatic system and aging. CNS Neurosci Ther. (2024) 30:e14803. doi: 10.1111/cns.14803, PMID: 38887168 PMC11183173

[49] Nguyen TT Ung TT Li S Lian S Xia Y Park SY . Metformin inhibits lithocholic acid-induced interleukin 8 upregulation in colorectal cancer cells by suppressing ROS production and NF-kB activity. Sci Rep. (2019) 9:2003. doi: 10.1038/s41598-019-38778-2, PMID: 30765814 PMC6376015

[50] Capraro M Pedrazzi M De Tullio R Manfredi M Cresta F Castellani C . Modulation of plasmatic matrix metalloprotease 9: A promising new tool for understanding the variable clinical responses of patients with cystic fibrosis to cystic fibrosis transmembrane conductance regulator modulators. IJMS. (2023) 24:13384. doi: 10.3390/ijms241713384, PMID: 37686190 PMC10488059

[51] Li R Zhao M Yao D Zhou X Lenahan C Wang L . The role of the astrocyte in subarachnoid hemorrhage and its therapeutic implications. Front Immunol. (2022) 13:1008795. doi: 10.3389/fimmu.2022.1008795, PMID: 36248855 PMC9556431

[52] Zang Y Jiang D Zhuang X Chen S . Changes in the central nervous system in diabetic neuropathy. Heliyon. (2023) 9:e18368. doi: 10.1016/j.heliyon.2023.e18368, PMID: 37609411 PMC10440454

[53] Du H Ma L Chen G Li S . The effects of oxyresveratrol abrogates inflammation and oxidative stress in rat model of spinal cord injury. Mol Med Rep. (2017) 17(3):4067–73. doi: 10.3892/mmr.2017.8294, PMID: 29257323

[54] Yu L-S Fan Y-Y Ye G Li J Feng X-P Lin K . Curcumin alleviates brain edema by lowering AQP4 expression levels in a rat model of hypoxia-hypercapnia-induced brain damage. Exp Ther Med. (2016) 11:709–16. doi: 10.3892/etm.2016.3022, PMID: 26997983 PMC4774356

[55] Kong G Xiong W Li C Xiao C Wang S Li W . Treg cells-derived exosomes promote blood-spinal cord barrier repair and motor function recovery after spinal cord injury by delivering miR-2861. J Nanobiotechnol. (2023) 21:364. doi: 10.1186/s12951-023-02089-6, PMID: 37794487 PMC10552208

[56] Sun X Dias L Peng C Zhang Z Ge H Wang Z . 40 Hz light flickering facilitates the glymphatic flow via adenosine signaling in mice. Cell Discov. (2024) 10:81. doi: 10.1038/s41421-024-00701-z, PMID: 39103336 PMC11300858

[57] Liu X Hao J Yao E Cao J Zheng X Yao D . Polyunsaturated fatty acid supplement alleviates depression-incident cognitive dysfunction by protecting the cerebrovascular and glymphatic systems. Brain Behav Immun. (2020) 89:357–70. doi: 10.1016/j.bbi.2020.07.022, PMID: 32717402

[58] Mueller SM McFarland White K Fass SB Chen S Shi Z Ge X . Evaluation of gliovascular functions of AQP4 readthrough isoforms. Front Cell Neurosci. (2023) 17:1272391. doi: 10.3389/fncel.2023.1272391, PMID: 38077948 PMC10701521

[59] Wang F Xu C Su C Li J Lin J . β-hydroxybutyrate attenuates painful diabetic neuropathy via restoration of the aquaporin-4 polarity in the spinal glymphatic system. Front Neurosci. (2022) 16:926128. doi: 10.3389/fnins.2022.926128, PMID: 35898407 PMC9309893

[60] Liu XL Ouyang FB Hu LT Sun P Yang J Sun YJ . Mesenchymal stem cells improve cognitive impairment and reduce Aβ Deposition via promoting AQP4 polarity and relieving neuroinflammation in rats with chronic hypertension-induced cerebral small-vessel disease. Front Aging Neurosci. (2022) 14:883503. doi: 10.3389/fnagi.2022.883503, PMID: 35663575 PMC9160459

[61] Jeon H Kim M Park W Lim JS Lee E Cha H . Upregulation of AQP4 improves blood–brain barrier integrity and perihematomal edema following intracerebral hemorrhage. Neurotherapeutics. (2021) 18:2692–706. doi: 10.1007/s13311-021-01126-2, PMID: 34545550 PMC8804112

[62] Haj-Yasein NN Vindedal GF Eilert-Olsen M Gundersen GA Skare Ø Laake P . Glial-conditional deletion of aquaporin-4 (*Aqp4*) reduces blood–brain water uptake and confers barrier function on perivascular astrocyte endfeet. Proc Natl Acad Sci USA. (2011) 108:17815–20. doi: 10.1073/pnas.1110655108, PMID: 21990350 PMC3203818

[63] Wang Y Huang J Ma Y Tang G Liu Y Chen X . MicroRNA-29b is a therapeutic target in cerebral ischemia associated with aquaporin 4. J Cereb Blood Flow Metab. (2015) 35:1977–84. doi: 10.1038/jcbfm.2015.156, PMID: 26126866 PMC4671118

[64] Salman MM Kitchen P Halsey A Wang MX Törnroth-Horsefield S Conner AC . Emerging roles for dynamic aquaporin-4 subcellular relocalization in CNS water homeostasis. Brain. (2022) 145:64–75. doi: 10.1093/brain/awab311, PMID: 34499128 PMC9088512

[65] Vasciaveo V Iadarola A Casile A Morello G Dante D Minotta L . Sleep fragmentation affects glymphatic system through the different expression of AQP4 in wild type and 5xFAD mouse models. Alzheimer’s Dementia. (2023) 19:e077763. doi: 10.1002/alz.077763, PMID: 36653878 PMC9850555

[66] Pisani F Simone L Mola MG De Bellis M Frigeri A Nicchia GP . Regulation of aquaporin-4 expression in the central nervous system investigated using M23-AQP4 null mouse. Glia. (2021) 69:2235–51. doi: 10.1002/glia.24032, PMID: 34038017 PMC8361696

[67] Zhang L Li D Yi P Shi J Guo M Yin Q . Peripheral origin exosomal microRNAs aggravate glymphatic system dysfunction in diabetic cognitive impairment. Acta Pharm Sin B. (2023) 13:2817–25. doi: 10.1016/j.apsb.2023.03.018, PMID: 37521866 PMC10372831

[68] Ikeshima-Kataoka H . Neuroimmunological implications of AQP4 in astrocytes. IJMS. (2016) 17:1306. doi: 10.3390/ijms17081306, PMID: 27517922 PMC5000703

[69] Su G Yu Z Liu G Zhang L Luo L Fang S . Icaritin promotes brain functional rehabilitation in ischemic stroke rats by regulating astrocyte activation and polarization via GPER. Free Radical Biol Med. (2025) 235:379–89. doi: 10.1016/j.freeradbiomed.2025.04.054, PMID: 40318814

[70] Lv T Zhao B Hu Q Zhang X . The glymphatic system: A novel therapeutic target for stroke treatment. Front Aging Neurosci. (2021) 13:689098. doi: 10.3389/fnagi.2021.689098, PMID: 34305569 PMC8297504

[71] Nishimura Y Masaki K Matsuse D Yamaguchi H Tanaka T Matsuo E . Early and extensive alterations of glial connexins, distal oligodendrogliopathy type demyelination, and nodal/paranodal pathology are characteristic of multiple system atrophy. Brain Pathol. (2023) 33:e13131. doi: 10.1111/bpa.13131, PMID: 36368713 PMC10154368

[72] Li T Chen X Zhang C Zhang Y Yao W . An update on reactive astrocytes in chronic pain. J Neuroinflamm. (2019) 16:140. doi: 10.1186/s12974-019-1524-2, PMID: 31288837 PMC6615111

[73] Kárpáti A Yoshikawa T Nakamura T Iida T Matsuzawa T Kitano H . Histamine elicits glutamate release from cultured astrocytes. J Pharmacol Sci. (2018) 137:122–8. doi: 10.1016/j.jphs.2018.05.002, PMID: 29858014

[74] Yuan Y Peng W Lei J Zhao Y Zhao B Li Y . AQP4 endocytosis-lysosome degradation mediated by MMP-9/β-DG involved in diabetes cognitive impairment. Mol Neurobiol. (2024) 61:8438–53. doi: 10.1007/s12035-024-04085-9, PMID: 38512439

[75] Vahsen BF Nalluru S Morgan GR Farrimond L Carroll E Xu Y . C9orf72-ALS human iPSC microglia are pro-inflammatory and toxic to co-cultured motor neurons via MMP9. Nat Commun. (2023) 14:5898. doi: 10.1038/s41467-023-41603-0, PMID: 37736756 PMC10517114

[76] Bihlmaier R Deffner F Mattheus U Neckel PH Hirt B Mack AF . Aquaporin-1 and aquaporin-4 expression in ependyma, choroid plexus and surrounding transition zones in the human brain. Biomolecules. (2023) 13:212. doi: 10.3390/biom13020212, PMID: 36830582 PMC9953559

